# Metabolic, Inflammatory, and Molecular Impact of Cancer Cachexia on the Liver

**DOI:** 10.3390/ijms252211945

**Published:** 2024-11-07

**Authors:** Daniela Caetano Gonçalves, Silvio Pires Gomes, Marília Seelaender

**Affiliations:** 1 Departamento de Biociências, Universidade Federal de São Paulo (UNIFESP), Santos 11015-020, Brazil; daniela.caetano@unifesp.br; 2 Instituto de Biociências (IBB), Departamento de Biologia Estrutural e Funcional, Anatomy Sector, Universidade Estadual Paulista (Unesp), Câmpus Botucatu, São Paulo 01049-010, Brazil; 3 LIM 26-HC, Departamento de Cirurgia, Faculdade de Medicina da Universidade de São Paulo, São Paulo 01246-903, Brazil

**Keywords:** cachexia, cancer, inflammation, liver, metabolism

## Abstract

Cancer-associated cachexia (CAC) is a severe wasting syndrome, marked by involuntary weight loss and muscle wasting. It is a leading cause of cancer-related morbidity and mortality, and is driven by systemic, chronic low-grade inflammation. Key cytokines, such as IL-6 and GDF15, activate catabolic pathways in many organs. This study examined the role of inflammation and metabolic disruption in the liver during CAC, focusing on its dual role as both a target and a source of inflammatory factors. The analysis covered protein and lipid metabolism disturbances, including the hepatic production of acute-phase proteins and insulin resistance. Hepatic inflammation contributes to systemic dysfunction in CAC. The increased production of C-Reactive Protein (CRP) impacts muscle wasting, while liver inflammation leads to insulin resistance and hepatic steatosis, aggravating the cachectic state. Therefore, understanding the molecular mechanisms of liver metabolism in CAC is essential for developing effective therapies. Potential interventions include anti-inflammatory treatments, anabolic strategies, and restoration of lipid metabolism. Further research is necessary to explore the liver’s full contribution to CAC and its systemic effects, allowing to the development of liver-targeted therapeutic strategies.

## 1. Introduction

Cancer-associated cachexia is a syndrome characterized by a marked and rapid decrease in body weight, primarily due to the depletion of skeletal muscle mass and white adipose tissue (WAT). This condition affects approximately half of all cancer patients and is present in the majority (over two-thirds) of those with advanced disease [1,2]. Cachexia is extremely detrimental and is considered the direct cause of 20–40% of all cancer-related deaths [3,4]. Cachectic patients also experience higher morbidity and diminished efficacy associated with radio- and chemotherapy treatments. This condition is often associated with malignancies of the pancreas, esophagus, stomach, lung, liver, and intestine [5,6].

Although the syndrome has garnered increasing attention in recent decades, its triggering cause remains unknown, and there is, to date, no therapy capable of reversing its harmful effects which compromise treatment, patient quality of life, and survival [7]. Weight loss in cachexia occurs involuntarily and results from the loss of muscle mass and adipose tissue, leading to a reduction in function, increased frailty, worsened treatment outcomes, and often death. [8]. As the incidence of cancer dramatically increases with age [3], it is expected that cachexia frequency will similarly increase. Indeed, cachexia is more prevalent in those cancer types that occur more frequently in older individuals, such as gastric cancer [9].

Cachexia is also associated with other diseases such as Congestive Heart Failure, Renal Failure, and chronic obstructive pulmonary disease, among other [10]. Typical symptoms of cachexia include marked loss of total body mass, anorexia, widespread inflammation, and pronounced muscle wasting [11]. The extensive muscle wasting also affects the thorax, diaphragm, and cardiac muscles; hence it is not surprising that most cancer deaths are related to respiratory failure [12] or to cardiac failure [13]. Given its impact on multiple organs, cachexia is a complex disease, and its severity is difficult to assess objectively. Indeed, it was only in 2011 that a diagnostic method was proposed and validated to stage the extent of cachexia [8].

Cachexia was proposed to be “characterized by involuntary and progressive weight loss, accompanied by muscle mass loss, which cannot be fully reversed by conventional nutritional support and leads to progressive functional decline”, and to encompass 3 stages [8].

Weight loss is the primary criterion for diagnosing cachexia. Specifically, a patient is considered cachectic if they are exhibiting weight loss greater than 5% of body weight within a 6-month period. Alternatively, patients with a Body Mass Index (BMI) lower than 20 kg/m^2^ are considered cachectic if exhibiting weight loss greater than 2%. Additionally, the presence of sarcopenia, which is low muscle mass, along with weight loss greater than 2%, also meets the diagnostic criteria [3].

Besides weight loss, cachexia is often accompanied by several additional symptoms that contribute to the overall decline in the patient’s condition. These symptoms include reduced muscle strength, which results in patients frequently experiencing decreased mobility and a diminished ability to perform daily activities; fatigue, as extreme and continuous tiredness is a common finding, further hindering routine activities; anorexia, leading to inadequate food intake and exacerbation of weight loss; and low albuminemia. These criteria provide a comprehensive framework for diagnosing cachexia, allowing for a more accurate and earlier detection, which is crucial for the implementation of appropriate therapeutic interventions [14].

Despite one of the main characteristics of cachexia being anorexia, the mode of tissue wasting greatly differs from that induced by [15]. It is important to note that treating anorexia through parenteral nutrition does not reverse cachexia, indicating that decreased caloric intake is not the primary cause of the disease [15]. Unlike starvation, in which adipose tissue is the first compartment mobilized, skeletal muscle loss occurs already with the onset of the disease [16]. However, although the primary tissue affected by cachexia is skeletal muscle, cachexia cannot be reduced to a muscle-wasting syndrome. Indeed, several other organs, such as the liver, heart, adipose tissue, the gut, and the central nervous system, are affected, rendering cachexia a true multi-organ syndrome [17].

Moreover, unlike chronic inflammatory diseases, cancer-related cachexia has a prompt onset, which is correlated with the speed of tumor growth and tissue infiltration. This characteristic distinguishes cancer-associated cachexia from other chronic conditions, making the clinical management and therapeutic interventions particularly challenging.

Our aim was to perform a narrative review on the impact of cachexia on the liver, focusing on its effects, underlying mechanisms, and potential therapeutic strategies.

Although cachexia is not restricted to cancer, as it is also associated, for instance, with COPD (chronic obstructive pulmonary disease), CVD (cardiovascular disease), and AIDS (acquired immunodeficiency syndrome), among others, we chose to solely address the cancer-related manifestation. Thus, this narrative review tackles only cancer patients and cancer cachexia models.

## 2. Literature Search Strategy

A literature search of observational studies was conducted to investigate the impact of cachexia and liver function and inflammation in cancer patients, and three literature databases were searched. With the help of a search string and considering this to be a narrative review, original scientific articles published in the last 20 years and indexed in Web of Science, PubMed, and LILACS were employed. No restrictions were applied to the initial electronic search. For the retrieval of studies, the following MeSH terms were used: “Cachexia” OR “Liver inflammation”. The goal was to gather evidence on the interplay between cachexia, liver dysfunction, and inflammation, providing a comprehensive understanding of how these factors influence the progression of cancer and potential treatment approaches.

## 3. Inflammatory Aspects of Cachexia

The etiology of cachexia is currently associated with chronic, low-grade systemic inflammatory process. This existing inflammatory process results from the interaction between the tumor and the host’s immune system, which then progresses to various organs such as the skeletal muscle, central nervous system, adipose tissue, pancreas, and liver. The pronounced catabolic process is thus, associated with the chronic release of inflammatory cytokines into the bloodstream and with organ and tissue inflammation [1].

It is proposed that the expression profile of cytokines released by the interaction between tumor cells, infiltrating macrophages, and tumor-associated macrophages (TAMs) is the determinant factor in triggering chronic systemic inflammation. The study highlighted the intricate relationship between the tumor, the host’s immune system, and various organs. In conclusion, cancer-associated cachexia is driven by a complex interplay of inflammatory and metabolic factors, profoundly impacting liver function [14].

Cytokines, particularly IL-6, play a crucial role in mediating the inflammatory response in cachexia. It was demonstrated that these cytokines can activate catabolic pathways in the liver and in the skeletal muscle, contributing to protein degradation [18]. The importance of cytokines in regulating hepatic metabolism during cachexia, with IL-6 being a primary mediator in the process, was also described [19]. Conversely, IL-4, an interleukin with anti-inflammatory properties, appears at lower expression levels in hepatocytes of cachectic cancer patients [20].

Inflammatory macrophages in the liver are associated with alterations in hepatic metabolism, contributing to insulin resistance and other metabolic dysfunctions in cachexia. The importance of these infiltrating cells in the progression of the syndrome is related with the production of inflammatory cytokines by the organ, which, after reaching the circulation, exacerbate muscle mass loss and protein degradation, worsening the symptoms of cachexia [21]. Additionally, it was noted that these infiltrating macrophages are associated with an increased production of pro-inflammatory cytokines such as TNF-α and IL-6, which can significantly alter liver function per se, aggravating cachexia [21]. Hepatic inflammation is linked to insulin resistance and metabolic dysfunction [21]. The inflammatory cytokines can induce the production of acute-phase proteins in the liver, as an adaptive response to inflammatory and metabolic stress, further exacerbating systemic inflammation and cachexia [16].

Tumor-associated macrophages (TAMs) within the tumor microenvironment. play a fundamental role in the maintenance and progression of cancer as they contribute to an environment conducive to tumor growth through promoting angiogenesis, metastasis, and resistance to drug treatment. These macrophages play an important role in the production of inflammatory mediators, contributing to the worsening of cancer-associated cachexia [22].

C-Reactive Protein (CRP) is a marker of the acute-phase response that shows significant increase during chronic inflammation [23]. Higher CRP levels in cachectic patients have frequently been reported [24]. Acute-phase proteins such as CRP are produced by the liver in response to inflammation, exacerbating cachexia in patients with liver cancer and other malignancies [1].

The presence of inflammatory serum proteins, such as CRP, can hence be employed in the diagnosis of cancer-associated cachexia. Increased CRP levels were reported as a sign of immune system activation in cancer [23], with important implications for the clinical management of this condition [1].

The effects of endurance training on PGE2 (prostaglandin E2, a pro-inflammatory eicosanoid) levels and carnitinepalmitoyltransferase (CPT) system activity were investigated in tumour-bearing rats. Exercise restored the activity of CPT I and CPT II enzymes in the liver mitochondria, probably by reducing PGE2 organ contents, and helped prevent hepatic steatosis. Moderate physical training was thus proved effective in mitigating the effects of cancer-associated cachexia [25].

Prostanoids, such as PGE2 also exacerbate muscle mass loss and anorexia [19]. Prostaglandin E2 (PGE2) has been implicated in promoting anorexia and muscle mass loss in animal models of cachexia. Negatively modulating protein synthesis in the liver and in the skeletal muscle [18].

PGE2 was identified as a potential target for therapeutic interventions due to its role in modulating inflammation and anorexia in animal models of cachexia [18], corroborating the study that demonstrated that exercise reduced the PGE2 content in the liver, reversing steatosis [25]. Similarly, strategies modulating IL-6 activity were suggested to be effective in reducing hepatic inflammation and the systemic effects of cachexia [19].

A study on Wistar rats investigated the effects of exercise and epinephrine on PGE2 production by Kupffer cells in the context of Walker 256 tumor-induced cachexia [26]. The study assessed the impact of physical exercise on hepatic mitochondrial CPT enzyme activity and PGE2 levels in cancer-bearing rats. The findings indicated that exercise restored enzymatic activity, reduced PGE2 levels, and helped prevent steatosis [26].

Another study investigated the effects of Walker 256 tumor-induced cachexia in Wistar rats, focusing on the infiltration of mononuclear cells into adipose tissue and the reduction in leptin concentration. The results showed an association between cell infiltration and decreased leptin levels, suggesting an inflammatory role in the modulation of this hormone during cachexia [23].

The activity of carnitinepalmitoyltransferase II (CPT II) in the liver mitochondria of rats bearing Walker 256 tumors was examined. The findings revealed a significant reduction in liver CPT II activity in cachectic rats, indicating that the presence of cachexia-inducing tumor had a negative effect on fatty acid oxidation in the liver. Indomethacin treatment was shown to partially reverse these effects, restoring CPT II activity. This study suggests that prostaglandins, particularly PGE2, may regulate CPT II activity in tumor-bearing rats [27].

Many studies have demonstrated an increase in inflammatory factors produced by the liver in the presence of cachexia in experimental models [25,28]. Hepatic inflammation is responsible for the increased production of acute-phase proteins by the liver, such as C-reactive protein (CRP) and serum amyloid A (SAA). Thus, the concentration of these proteins in the blood is directly related to IL-6 production by the liver and systemic inflammation, which is inversely proportional to patient survival span [26]. Prostaglandins, especially type 2 (PGE2), are potent paracrine mediators of the local inflammatory response. Additionally, PGE2 can induce the synthesis of TNF-α, IL-6, and IL-1 in macrophages and other cell types [29].

Despite previous findings showing the occurrence of hepatic inflammation, there are few studies in the literature indicating that this occurs in human cachexia [30]. The study analyzed liver samples from patients (with and without cachexia) who underwent surgery for pancreatic ductal adenocarcinoma (PDAC), to investigate the role of the liver in the induction and regulation of cachexia. Analyses were conducted on the interaction between peripheral blood mononuclear cells, histiocytes, and liver cells of these patients. The authors found a significant increase in the number of CD-68-positive monocytes associated with increased IL-6 levels, when compared to liver samples from cancer patients without cachexia. Based on this information, the authors suggested that the liver plays a significant role in the development of cachexia, and hepatic parenchymal cells can be activated by macrophage-mediated signaling to produce pro-inflammatory cytokines such as IL-6 [30]. Another study showed similar findings in cachectic rodents and humans, demonstrating a progressive increase of CD-68-positive myeloid cells in the liver as cachexia develops [31].

The enzymatic activity and gene expression of the mitochondrial carnitinepalmitoyltransferase enzyme complex are reduced in the liver of animals with cancer cachexia, as previously reported by our group [25,32,33]. The disruption of this enzyme complex hinders the entry of fatty acid into the cell mitochondria, compromising the formation of ATP, thus interfering with hepatic energy metabolism, and increasing the content of intracellular fatty acids, leading to hepatic steatosis [34].

In the hepatic acute-phase response, cytokines bind to specific surface receptors on target cells: three main groups of cytokines have been identified: those that act essentially as positive or negative growth factors for a variety of cells; cytokines with pro-inflammatory properties (TNF-α/β, IL-1α/β, IL-6, IFN-α/γ, IL-8, and macrophage inhibitory protein); and factors with anti-inflammatory activity [35].

Hepatocytes exhibit a variety of receptors for pro-inflammatory cytokines, including receptors for IL-1α, IL-1β, TNF-α, IL-6, and PGE. Non-parenchymal liver cells also have the ability to synthesize a variety of cytokines [36]. Kupffer cells (resident tissue macrophages) synthesize the pro-inflammatory cytokines IL-1, IL-6, and TNF-α in response to phagocytosis or to the binding of compounds such as endotoxin; these cells can also be induced by the cytokine IFN-γ. Similarly, under these conditions, chemokines are released by Kupffer cells to mediate the migration of blood neutrophils and monocytes [35].

The expression of many of the pro-inflammatory cytokines is regulated by the nuclear factor kappa B (NF-κB) transcription pathway [37]. In experimental models, the NF-κB signaling pathway contributes to the development of cancer-associated cachexia [38], and in patients, a recent study found that the expression of the NF-κB p65 subunit was 25% higher in the skeletal muscle of those with gastric cancer compared to the controls [37]. However, one study did not observe any statistical differences in NF-κB pathway protein expression over an extended period during the development of cachexia in rodents, suggesting that NF-κB may not play a role in liver inflammation [31]. Nonetheless, the same study showed an increase in the expression of proteins from the inflammasome pathway in the liver of cachectic animals [31].

Inflammation in cachexia is systemic, and many studies have investigated strategies to reduce whole body inflammation. To that end, supplementation with omega 3 fatty acids, HMB, L-carnitine, and other nutrients have been studied, as well as COX-inhibiting drugs. Although the results with animal models show effects, the literature on patients does not provide conclusive evidence.

Table 1 below presents the inflammatory profile produced by the liver in cancer-induced cachexia.

## 4. Hepatic Metabolic Alterations in Cachexia

To clearly define the molecular and metabolic determinants of tissue wasting, it is essential to employ a systemic approach to delineate the contribution of each individual organ in the cachectic process and to understand the role of the tumor in this process and the interaction between the two compartments. Indeed, while cachexia is a metabolic disorder characterized by tissue wasting, resistance to anabolic signals, and an overall catabolic state, cancers, on the other hand, are highly proliferative and energy-demanding tissues [38,39]. Consequently, the metabolic alterations present in cachectic patients result in negative energy balance and in the release of metabolites into the bloodstream, further supporting tumor growth [40].

## 5. Hepatic Alterations in Protein Metabolism

Cachexia induces pronounced muscle protein degradation, resulting in a substantial release of amino acids, such as alanine and glutamine, into the bloodstream. The released glutamine is extensively utilized by the tumor both as an energy substrate and as a nitrogen source for cell growth, while a significant portion of the alanine is used by the liver to increase hepatic protein synthesis [17].

Under these conditions, there is a significant shift in hepatic protein metabolism; despite a decrease in the production of hepatic proteins like albumin- there is a considerable increase in positive acute-phase response (APR) proteins [19]. Alongside the increase in C-reactive protein (CRP), animal models of cancer cachexia also show augmented expression of serum amyloid A, α1-antitrypsin, α1-acid glycoprotein, fibrinogen, and complement factors B and C3 [19].

The increased production of CRP is associated with the production of complement factors that are related to immune regulation, such as enhanced phagocytosis and prevention of platelet aggregation, among other [19].

The α1-acid glycoprotein is also related to increased phagocytosis, the inhibition of platelet aggregation, and a possible relation to the spacing of collagen fibers. Haptoglobin is associated with the removal of hemoglobin from the plasma. The positive acute-phase proteins produced and released by the liver are important inflammatory factors in cancer cachexia. CRP is an important marker used for the diagnosis and prognosis of cachexia. Serum amyloid A appears to have a synergistic action with interleukin-6, stimulating muscle proteolysis, thereby perpetuating the cycle of amino acids and proteins between the muscle and liver [41].

## 6. Hepatic Alterations in Lipid Metabolism

Lipids are the main energy substrate for hepatocytes, which store glucose that has been taken up, as glycogen, and contribute to control of glucose homeostasis.

Changes in lipid metabolism in cancer cachexia induce a marked reduction in the total adipose tissue fat mass and increase in lipolysis, fatty acid oxidation, and hyperlipidemia. Furthermore, cachexia is associated with a reduction in the lipogenesis rate and a decrease in the activity and expression of lipoprotein lipase (LPL) [35,42,43]. Increased mRNA and protein levels of hormone-sensitive lipase (HSL) can be detected in tissues of cachectic patients (notably in the liver and white adipose tissue) [44]. The reduction in white adipose tissue content, a consequence of lipodystrophy and lipoatrophy, is also a feature of cancer cachexia; in fact, an 85% reduction in body fat has been reported in patients with lung cancer. This often leads to hyperlipidemia and insulin resistance, as well as complications in antitumor therapies [45]. The wasting of fat deposits cannot be fully explained by a reduced appetite leading to anorexia, and often occurs in association with cancer cachexia [46]. Some studies [44,45,47,48] have proposed that the progression of cachexia is closely related to the imbalance of catabolic and anabolic processes in peripheral tissues, notably in the adipose tissue.

A recent study showed that in murine models of cachexia, there is a considerable increase in enzymes involved in ceramide synthesis in the liver of cachectic animals. As this increase was not identified in muscle and adipose tissues, the liver is believed to be the major contributor to ceramide turnover and the increased sphingolipid levels in the syndrome [49].

Previous studies conducted by our group have demonstrated that in animal models of cachexia, hepatic function is significantly compromised, particularly in relation to lipid metabolism in rats bearing Walker 256 tumors. In the first study, it was shown that hepatocytes from tumor-bearing rats exhibited higher content of triacylglycerol (TAG) in very-low-density lipoproteins (VLDLs) and greater presence of lipid droplets compared to the control group. These findings indicated the development of hepatic steatosis in cachectic animals [50].

Hepatic steatosis is a pathological condition of the liver that can be associated with several factors: decreased lipid oxidation, increased uptake and re-esterification of fatty acids, elevated de novo lipogenesis, or impaired VLDL secretion. In the context of cancer cachexia, these processes are significantly altered [50]. Specifically, with respect to lipid oxidation, another study by our group [50] showed disruption of hepatic mitochondrial carnitinepalmitoyltransferase (CPT) enzyme complex inserted in the mitochondrial membranes, which is essential for the transport and oxidation of long-chain fatty acids in mitochondria. This study found a 56% reduction in CPT II activity in cachectic rats, highlighting a profound impairment in hepatic fatty acid oxidation capacity. This does not only contribute to the accumulation of lipid droplets in the liver but also exacerbates the overall metabolic dysfunction observed in cachexia, rendering the organ uncapable of obtaining ATP at an appropriate range.

These findings underscore the multifactorial disruption of hepatic lipid metabolism in cancer cachexia, where both lipid accumulation and impaired oxidative processes lead to the development of hepatic steatosis [50].

Another study demonstrated a reduced capacity for ketone body production by the liver of Walker 256 tumor-bearing rats: and disruption of hepatocyte zonation patterns [51].

Regarding the distribution of CPT I and II mitochondrial enzymes in hepatocytes CPT II was found, in the cachectic animal liver to be more abundant in the perivenous zone of the liver compared to the periportal zone, unlike in normal physiological states, and there was also, a reduction in CPT I activity in these cells [32]. A study indicated an increase in plasma TAG and VLDL-TAG in cachectic rats compared to the control group [32]. In contrast, there was a decrease in hepatic VLDL secretion, indicating a possible reduction in VLDL production by the liver. These results were accompanied by an analysis of the expression of ApoB and MTP, proteins related to VLDL assembly, which showed a decrease in mRNA levels in the tumor-bearing group compared to the control group [25].

**Figure 1:**
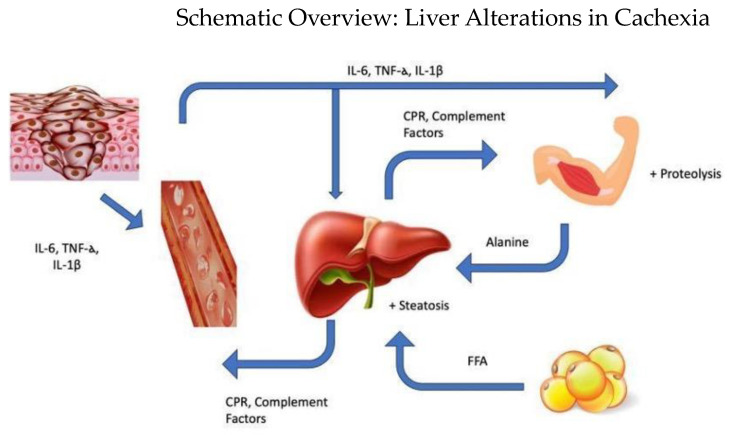
Schematic overview of the liver’s central role in the development of cachexia. The Liver interacts with the immune system, muscle tissue, and adipose tissue in cycles involving inflammation, proteolysis, and fat mobilization. The figure was created using resources from BioRender (https://www.biorender.com), accessed on 15 October 2024.

These studies have shown that in tumor-bearing animals, hepatic steatosis is related to decreased lipid oxidation in the liver and decreased VLDL assembly and secretion, leading to greater hepatic lipid storage. This profile exacerbates the cachectic condition, as hepatic lipid accumulation leads to cellular toxicity, impairing function and worsening the metabolic chaos present in the organism. The reduction in PGE2 production in the liver leads to the reestablishment of lipid metabolism parameters, which was demonstrated in [28]. Nevertheless, scientific literature demonstrating liver inflammation in patients with cancer-associated cachexia is still scarce [28]. One of the explanations for liver alterations in cachexia. Finally, the alteration of cardiolipins in hepatic mitochondria, mediated by TNF-alpha, impairs ATP production from fatty acids, as shown in Figure 1. 

## 7. Pathophysiology of Cancer-Associated Cachexia, Inflammation, and Drug Resistance

As described above, cachexia syndrome can compromise cancer treatment, both because of the change in nutritional status and malnutrition that can intensify during chemotherapy treatment and also, owing to drug resistance as a result of the metabolic changes. The changes described in the liver are important and contribute negatively to the pharmacokinetics of drugs, in addition to the other changes observed in the intestine, skeletal muscle, adipose tissue, and central nervous system [52]. The first step includes absorption (A), which may be compromised in cachexia due to altered microbiota and an impairment of the intestinal barrier [53]. As most of the drugs used in treatment are also responsible for altering the intestinal microbiota, during treatment, there is a progressive alteration of the intestinal barrier, impairing drug absorption.

After absorption (A), the drug is distributed (D) throughout the body. Distribution may be altered during the syndrome, as albumin is the main protein involved in the transport of many substances through the blood and with the progression of cachexia, patients show a considerable decrease in albuminemia, as described before. In addition, muscle and adipose tissues are important in drug distribution and are also reduced in terms of mass [52]. Drug metabolization (M) is the third step and is related to the amount given and clearance patterns. The liver also plays a crucial role in this stage, since a large part of drug metabolism takes place in this organ and during cachexia, there is a significant change in the expression of enzymes responsible not only for transporting but also for metabolizing drugs, such as CY3A4, which is responsible for metabolizing xenobionts. The last stage is the excretion (E) of the drugs by the kidneys. Kidney function can be compromised in cachexia due to inflammation, but also as a reflex of the organ’s important role during chemotherapy [54].

## 8. Conclusions and Future Directions

The liver plays a crucial role as a manager of intermediary metabolism and is directly affected by systemic inflammation, resulting in significant metabolic alterations in cancer-associated cachexia. The disruption of hepatic lipid metabolism decreases ATP production, promotes increased futile energy expenditure, and can lead to non-alcoholic hepatic steatosis. The increased synthesis of acute-phase proteins exacerbates the inflammatory condition and muscle catabolism and augments the requirement of the liver for alanine and other amino acids, that has to be met by protein breakdown in the muscle. Despite its importance and contribution to the syndrome of cachexia, few human studies are available on the role of the liver in cachexia.

Cancer-associated cachexia lacks consensus treatment; the proposed therapies include physical exercise, anabolic medication, and anti-inflammatory supplements that can mitigate systemic inflammation. Additionally, strategies that promote better lipid oxidation in the liver are necessary to prevent dyslipidemia and hepatic steatosis. Whatever the path chosen for mitigating the disease, it must consider the role and relevance of the liver.

Despite the fact that nutritional intervention is well-characterized in the management of cachexia, the effectiveness is limited in cancer patients due to the complexity of the metabolic state and owing to anabolic resistance. Beyond nutrition, additional therapeutic approaches have been explored, such as the use of pharmacological agents (anti-inflammatory drugs, metabolic modulators, and hormone receptor agonists), which aim to mitigate systemic inflammation and preserve muscle mass. However, the development of effective treatments remains a challenge, and there is an increasing need for research to identify multifactorial interventions capable of addressing the underlying mechanisms of cachexia more effectively. For that purpose, liver function must be seriously taken into account.

## Figures and Tables

**Table 1:** Inflammatory profile produced by the liver in cancer-induced cachexia.

Inflammatory Mediator	Producing Cells	Function	Levels in Cancer Cachexia
Interleukin 6 (IL-6)	Infiltrated Macrophages, TAMs, CD-68-Postive Monocytes	Muscle proteolysis, insulin resistance, increase in CRP, systemic inflammation	increased
Interleukin 1β (IL-1β)	Infiltrated Macrophages, TAMs, CD-68-Postive Monocytes	Muscle proteolysis, insulin resistance, increase in CRP, systemic inflammation	increased
Interleukin 4 (IL-4)		Anti-inflammatory properties	decreased
Interleukin 8 (IL-8)	Infiltrated Macrophages, TAMs, CD-68-Postive Monocytes	Pro-inflammatory properties, systemic inflammation	increased
Tumor Necrosis Factor α (TNF-α)	Infiltrated Macrophages, TAMs, CD-68-Postive Monocytes	Muscle proteolysis, insulin resistance, increase in CRP, systemic inflammation	increased
Interferon γ (IFN-γ)	Infiltrated Macrophages, TAMs, CD-68-Postive Monocytes	Pro-inflammatory properties, systemic inflammation	increased
C-Reactive Protein (CRP)	Hepatic Cells	Immune system activation	increased
Prostaglandin E2 (PGE 2)	Kupffer Cells	Muscle mass loss, anorexia, decreased protein synthesis in liver and muscle, liver steatosis, decreased CPT II maximal activity	increased
